# Burden of nosocomial infections in intensive care units: Cost of antibiotics, the extra length of stay and mortality rate

**DOI:** 10.22088/cjim.15.3.478

**Published:** 2024-08-01

**Authors:** Masoome Zolfaghari, Arash Seifi, Ebrahim Jaafaripooyan, Zahra Jahangard-Rafsanjani, Shirin Afhami, Mostafa Mohammadi, Mohammad Masoud Emami Meybodi, Mohammadreza Salehi, Esmaeil Mohammadnejad

**Affiliations:** 1 Department of Radiology, Iran University of Medical Sciences, Tehran, Iran; 2 Department of Infectious Diseases, Research Center for Antibiotic Stewardship and Antimicrobial Resistance, Imam Khomeini Hospital, Tehran University of Medical Sciences, Tehran, Iran; 3 Department of Health Management and Economics, School of Public Health, Tehran University of Medical Sciences, Tehran, Iran; 4 Department of Clinical Pharmacy, School of Pharmacy, Tehran University of Medical Sciences, Tehran, Iran; 5 Department of Infectious Diseases, School of Medicine, Tehran University of Medical Sciences, Tehran, Iran; 6 Department of Anesthesiology and Critical Care, School of Medicine, Tehran University of Medical Sciences, Tehran, Iran; 7 Department of Radiology, Tehran University of Medical Sciences, Tehran, Iran; 8 Department of Medical-Surgical Nursing, Research Center for Antibiotic Stewardship and Antimicrobial Resistance, Tehran University of Medical Sciences, Tehran, Iran

**Keywords:** Healthcare-associated infections, ICU, Economic burden

## Abstract

**Background:**

Healthcare-associated infections (HAIs) in intensive care unit (ICU) patients significantly complicate the normal hospitalization process and affect patients’ condition, length of hospitalization, mortality, and treatment cost. In this study, we aimed to determine the prevalence and economic burden of HAIs.

**Methods:**

The study involved all patients with a confirmed HAIs (based on CDC/NHSN case-definitions); in the general ICU of a tertiary university hospital in Tehran, from April 2020 to March 2021. The patients’ information, including length of hospitalization, outcome, type and cost of prescribed antibiotics, were recorded.

**Results:**

During the study period, 119 HAIs were found in 1395 (43% F / 57% M) patients. The prevalence of nosocomial infections was 8.53%. The mean duration of hospitalization in all ICU patients was 4.7 ± 3.1 days, and 31.85 ± 18.96 days in patients with HAIs. The most common organisms involved in HAIs are *Acinetobacter baumannii* (54.6%), *Klebsiella pneumoniae* (30.3%), *E. coli* (15.1%), and Enterococcus spp. (12%). Incidence density of ventilator-associated pneumonia (VAP), central line-associated bloodstream infection (CLA-BSI), and catheter-associated urinary tract infection (CA-UTI) per 1000 device-days were 36.08, 17.57, and 8.86, respectively. The total cost of antibiotics for HAIs was € 105,407. Among these, the highest consumption costs were for carbapenems, followed by colistin and caspofungin.

**Conclusion:**

This study showed the high burden of nosocomial infections in ICUs. Strategies for more strict infection prevention and control are necessary to reduce this burden.

Nosocomial infections, also named healthcare-associated infections (HAI), are infection(s) not present at admission but are acquired during the process of medical care in any health care facility or emerge after discharge. The Center for Disease Control and Prevention (CDC) categorized the types of HAI as central line-associated bloodstream infections (CLABSI), catheter-associated urinary tract infections (CAUTI), surgical site infections (SSI), and ventilator-associated pneumonia (VAP) (1). According to the World Health Organization (WHO), approximately 15% of all hospitalized patients suffer from HAIs (2). 

HAIs increase morbidity, mortality, and cost burden in hospitalized patients, especially in precarious settings such as intensive care units (ICU). The prevalence of HAIs in ICU is 3 to 5 times higher than in other care units, and its incidence rate is 50% in developing countries, almost two-fold of the industrialized nations (3). The ICU department compromises about 10% of hospital beds, but HAI episodes in the ICU account for 20% of all (4).

The severity of illness, physiological and psychological stress, sleep deprivation, older age, more extended hospitalizations, and malnutrition alter ICU admitted patients' immune systems and make them susceptible to HAIs. HAIs impose a significant clinical and economic burden on the health systems of all countries. HAI's annual direct medical costs in the USA hospitals have ranged from $28.4 to $33.8 billion and 16 million extra hospitalization days (5). The burden of HAIs in non-developed countries is less studied. In Argentina's ICUs, the additional cost for VAP is estimated at $2,255 (6). WHO reports the prevalence of HAIs in Iran to be 8.8% (2), with a 14.8% mortality rate due to these infections. It is estimated that Iranian patients spend $4.74 million annually on HAI incurred costs (7). 

HAIs impose exorbitant costs on the health system and patients. Few studies regarding this critical issue have been conducted in Iran, so further studies are needed to understand this problem's dimensions to adopt solutions. In this study, we aimed to determine the prevalence and economic burden of HAIs in the general ICU of a tertiary university hospital in Tehran, Iran. 

## Methods

This cross-sectional, study was conducted from April 2020 to March 2021 in the general ICU of the Tehran Imam Khomeini Hospital, a tertiary university hospital. All admitted patients to the general ICU during this time frame with a confirmed diagnosis of HAIs; and willing to participate in the investigation entered the study. We excluded the participants whose medical records were incomplete in terms of demographic characteristics, definite diagnosis, medications, and outcome. The study is approved by the Ethics Committee of Tehran University of Medical Sciences and Iran National Committee for Ethics in Biomedical Research with Ethics Code IR.TUMS.IKHC.REC.1397.147 as medical student dissertation.

We collected the medical information of the participants using a checklist designed based on similar studies. The checklist items included the demographic characteristics of the patients (age, sex, date of admission, date of death or discharge), length of hospitalization, type of specimen sent for culture, type of organism, HAI type (CLABSI, CAUTI, VAP), outcome (discharge or death), type of medication, duration of treatment, and medication cost. We investigated the patients' hospital medical records, detection forms available in the archive of the hospital’s Infection Control Unit, pharmacy documentation in terms of medications, and their costs to gather and record the data. The HAI diagnosis was based on the patients’ clinical symptoms, culture results, and CDC/NHSN criteria (1). The diagnosis was further revised and confirmed by infectious disease specialists.

 The DAIs' diagnostic criteria were as follows. CLABSI was defined as: central line was in place for >2 calendar days (it was in place on the date of blood sampling or the day before);and having a positive blood culture of a known BSI pathogen (such as Staphylococcus aureus, Klebsiella pneumoniae, and etc.) in at least one blood sample such that the growing organism was not related to infection in another site; or at least one of the fever, chills, or hypotension; in addition, common commensals (such as Staphylococcus epidermidis) were cultured at least from two blood samples drawn on separate occasions. CAUTI was defined as: an indwelling urinary catheter in place for >2 days (it was in place on the date of urine sampling or the day before); and fever (T >38℃), urgency, frequency, dysuria, suprapubic tenderness, or costovertebral angle pain/tenderness with a positive urine culture of ≥105 CFU/mL of no more than two isolated species. And finally, VAP defined as: at least 20% increase in the minFiO2 or a minimum increase of 3 cm-H2O in the PEEP (positive end-expiratory pressure) to maintain oxygenation for a sustained period of more than 2 days; and it happened in the setting of an infection (fever, leukocytosis, etc.) and antibiotics are instituted for a minimum of 4 days; and the detection of respiratory pathogens on cultures or by equivalent techniques (such as Polymerase Chain Reaction (PCR)).

To assess the economic burden of HAIs, we evaluated the instrument/day and patient/day registration forms, which are completed daily by ICU nurses. The total number of patient/day, instrument/day, and also by detail ventilator/day, urinary catheter/day, and temporary central venous catheter/day were recorded during the study period. We assessed each patient's medication costs using the hospital information system's (HIS) data and the hospital pharmacy’s data bank.

To evaluate the outcome of patients diagnosed with HAIs, the total number of patients admitted to the ICU during the study time frame, their mean age, gender ratio, and the number of deaths and discharges were recorded. The mean age, gender ratio, relative frequency of organisms, relative frequency of outcomes, the total cost of medication, the incidence of HAIs per 1000 instrument/days, the total number of HAI episodes and divided (VAP, CLABSI, CAUTI), and the number of instrument/days were recorded and evaluated. We used IBM™SPSS®20 for statistical analysis of the data. The level of significance is considered p<0.05. Relevant tests measured data distribution (normal or non-normal). Based on the normality test results, the t-test or chi-square test were used accordingly. 

## Results

A total of 1395 patients were admitted to the General Intensive Care Unit of Imam Khomeini Hospital from April 2020 to March 2021. The mean age of the patients was 59.2 years (SD=23.6). Out of 1395 patients admitted to the ICU, 119 (8.53%) had nosocomial infections. The demographic characteristics of all the patients are shown in table 1. The mean length of hospitalization in all ICU patients was 4.7 days (SD=3.1). In comparison, the mean length of hospitalization in ICU admitted patients with nosocomial infections was 31.85 days (SD=18.96), which was significantly higher than the average length of hospitalization of ICU patients (p<0.05).

**Table 1:** Demographic characteristics of the participants

	**Patients**		**Mean age (year)**	**Mean hospitalization length (day)**	**Outcome**	
	**Female (%)**	**Male (%)**			**Death (%)**	**Discharge (%)**
**All ICU-admitted patients**	1395		59.2	4.7	165 (11.8)	1230 (88.2)
	600 (43)	795 (57)				
**ICU-admitted patients with HAI**	119		58.83	31.85	64 (53.8)	55 (46.2)
	49 (41.2)	70 (58.8)				

Table 2 demonstrates the prevalence of all organisms isolated from ICU-admitted patients with HAIs. It should be mentioned that some patients were infected with more than one organism. The three most common organisms responsible for HAIs in our patients were Acinetobacter, Klebsiella pneumonia, and E.coli. 

**Table 2:** Causative organisms in ICU’s infections

**Organism**	**VAP**	**CAUTI**	**CLABSI**	**Number**	**Percent**
**Acinetobacter baumannii**	49	5	11	65	54.6
**Klebsiella pneumoniae**	22	7	7	36	30.3
**E.coli**	8	8	2	18	15.1
**Enterococcus spp.**	0	3	12	15	12
**Candida albicans**	0	10	3	13	10.9
**Pseudomonas aeruginosa**	6	5	1	12	10
**Staphylococcus aureus**	2	0	6	8	6.7
**Non-albicans candida**	0	6	1	7	5.8
**Enterobacter cloacae**	0	0	2	2	1.6
**Staphylococcus epidermidis**	0	0	1	1	0.8
**Streptococcus agalactiae**	0	0	1	1	0.8
**Streptococcus pneumoniae**	1	0	0	1	0.8
**Proteus spp.**	0	1	0	1	0.8

CAUTI: Catheter-associated Urinary Tract Infection; CLABSI: Central Line-associated Blood Stream Infection; VAP: Ventilator-associated Pneumonia.

The total cost of medications used to treat HAIs in the general ICU was about 105,407.7 euros during our study time frame. Table 3 shows the type and amount of antibiotics used to treat bacterial HAIs among our study population, as well as their cost. Among the antibiotics used in the general ICU to treat HAIs, the highest amounts were related to Carbapenems (8258 vials), Colistin (6349 vials), and Glycopeptide antibiotics (4750 vials), including teicoplanin and vancomycin. 

As mentioned before, some of our patients suffered from more than one HAI. In total, 180 episodes of HAIs happened among 119 ICU-admitted patients. The percentage of infection based on hospitalization is 12.90% among all 1395 patients admitted to the general ICU during our study. The total patient day is 5330, and the incidence of infection in 1000 patient-days is 33.7. Table 4 shows the distribution of HAIs among the ICU admitted patients. 

**Table 3:** Distribution of antibiotic costs in patients with HAIs

**Antibiotic**	**Vials (number)**	**Price per vial (euros)**	**Total cost (euros)**
**Carbapenems**	8258	4.46	39,686.43
**Colistin**	6349	2.73	17,316.25
**Glycopeptides**	4750	7.04	6,391.78
**Metronidazole**	2791	2.73	7,612.69
**Fluoroquinolones**	2550	1.64	6,629.06
**Cotrimoxazole**	1643	0.36	597.56
**Ampicillin/Sulbactam**	1113	0.73	809.31
**Piperacillin/Tazobactam**	945	5.45	5,152.28
**Clindamycin**	763	0.54	416.19
**Cephalosporins**	757	1.03	627.11
**Aminoglycosides**	653	0.24	176.22
**Linezolid**	173	15.44	2,671.08
Anti-fungals*	-	-	17321.74
**TOTAL**			105,407.70

**Table 4:** Distribution of HAI types in patients

	**Infection episodes (number)**	**Device-Days**	**Incidenc of infection in 1000 Device-Days**	**Device use ratio**
**CAUTI**	46	5192	8.86	0.97
**CLABSI**	42	2390	17.57	0.45
**VAP**	92	2550	36.08	0.48

CAUTI: Catheter-associated Urinary Tract Infection; CLABSI: Central Line-associated Blood Stream Infection; VAP: Ventilator-associated Pneumonia.

## Discussion

HAI is a major problem in the health systems of all countries. The prevalence of HAIs in healthcare facilities depends on many factors, such as medical interventions and patients’ characteristics. In studies conducted regarding this issue in Iran, the HAI rate is reported to be very variable. In this study, we evaluated the prevalence of three device-associated healthcare infections and the economic burden of their treatment in the general ICU of a tertiary university hospital in Tehran. 

The prevalence of HAIs in our study was found to be 8.53%, with VAP accounting for 51%, CAUTI 26%, and CLABSI 23% of all HAI episodes. The mortality rate among ICU-admitted patients with HAI was 53.8 in our study population. We found the three most common microorganisms responsible for HAIs: Acinetobacter, Klebsiella pneumoniae, and E. coli. The total medication cost of HAIs in our study duration is 105,407.7 euros, with 84% being accounted for antibiotic costs. The highest amount of antibiotics used in the general ICU for the treatment of HAIs were Carbapenems. 

HAIs prevalence in our study (8.53%) is in line with the infectious disease reference book that mentions the HAI prevalence between 5-20% (8). Studies in the United States and Italy have reported the prevalence of HAIs to be 18% and 30.4%, respectively; the lower prevalence of HAIs in our center might be due to less invasive medical procedures and less use of deep catheters (9, 10). The hospital’s infection control unit’s efforts in recent years have resulted in the staff’s obligation to comply with health regulations and lessen HAI prevalence. The most common HAI in the present study is VAP (51%), which complies with the results of studies done in Mexico (39.7%), Italy (45.5%), the US (64%), and India (29.5%), in all of which pneumonia is the most common HAI reported (9-12). In a recent study conducted in 491 hospitals of Iran, the most common HAIs have been CAUTI and VAP, respectively (13).

The microorganisms most responsible for HAIs have changed during the last three decades. Staphylococcus aureus in the 1950s, gram-negative bacterias in the 1970s, and gram-positive cocci in the 1980s showed resistance to antibiotics and were the most common strains isolated from patients with HAIs. In our study, the most common bacterial strain isolated was Acinetobacter (36.1%), followed by Klebsiella pneumonia (20%) and E.coli (10%). Our findings regarding microorganisms are harmonious with the study conducted by Afhami et al., which found Acinetobacter (33.5%) and Klebsiella (19%) to be the most common responsible microorganisms for HAIs in Tehran's university hospitals (14). In a study done in a university hospital in Kashan, Iran, the most reported strains were Acinetobacter, Klebsiella pneumoniae, and E-coli, with the most common HAI to be VAP (15). 

In this study, the most commonly used antibiotics to treat HAIs were reported to be Carbapenems, followed by Colistin and Vancomycin. In a study on the antibiotics used in Latin American ICUs, Carbapenem was the most widely used alone or in combination with Vancomycin or other antibiotics (16). The medication cost for treating HAIs in the general ICU of Tehran Imam Khomeini Hospital in 2017 was 105,407.7 euros. The whole economic burden imposed by HAIs on the health system, such as increased hospitalization length, personnel, and consumable costs, are more than that. It should be noted that HAIs impose a significant burden on the patients and their families as well. The annual economic burden of HAIs in the US and Europe is $ 6.5 billion and € 7 billion, respectively (2, 17). 

In our study, the length of hospitalization among patients with HAIs is significantly longer than the rest of ICU admitted patients. In a study by Wakefield et al., the average length of hospitalization in patients with HAI was 18 days longer than other patients (18). About 53.8% of our ICU admitted patients with HAI died. This study could not determine whether the cause of death was the underlying disease or the HAI, but the additional infection could be the facilitator of the patient’s outcome. Although there are many confounders to compare the crude death and discharge ratios of ICU admitted patients with and without HAIs, it can be roughly said that the mortality rate is higher among patients with HAIs. In a Tunisian hospital study, 50% of deaths in the ICU were associated with HAIs (19). HAIs are reported to be the direct cause of 37000 deaths in Europe and 99000 deaths in the US annually (2, 20). 

There were some limitations for this study including lack of characteristics of patients, underlying diseases and risk factors for infections, as well as, appropriate or inappropriate prescription of antibiotics (antibiotic stewardship) that could be the objectives of further studies.

This study showed the high burden of nosocomial infections in ICUs. Complete elimination of HAIs has not been possible yet, but successful HIAs prevention requires comprehensive attention to control of infection sources and the application of appropriate infection prevention measures such as the faster detection of infections, trying to find the causative microorganisms, implementation of infection prevention principles, antimicrobial stewardship program, and education of hospital staff.
